# Digital Information Ecosystems in Modern Care Coordination and Patient Care Pathways and the Challenges and Opportunities for AI Solutions

**DOI:** 10.2196/60258

**Published:** 2024-12-02

**Authors:** You Chen, Christoph U Lehmann, Bradley Malin

**Affiliations:** 1 Department of Biomedical Informatics Vanderbilt University Medical Center Nashville, TN United States; 2 Department of Computer Science Vanderbilt University Nashville, TN United States; 3 Clinical Informatics Center University of Texas Southwestern Medical Center Dallas, TX United States; 4 Institut für Medizinische Informatik Universitäts Klinikum Heidelberg Heidelberg Germany; 5 Department of Biostatistics Vanderbilt University Medical Center Nashville, TN United States

**Keywords:** patient care pathway, care journey, care coordination, digital information ecosystem, digital technologies, artificial intelligence, information interoperability, information silos, workload, information retrieval, care transitions, patient-reported outcome measures, clinical workflow, usability, user experience workflow, health care information systems, networks of health care professionals, patient information flow

## Abstract

The integration of digital technologies into health care has significantly enhanced the efficiency and effectiveness of care coordination. Our perspective paper explores the digital information ecosystems in modern care coordination, focusing on the processes of information generation, updating, transmission, and exchange along a patient’s care pathway. We identify several challenges within this ecosystem, including interoperability issues, information silos, hard-to-map patient care journeys, increased workload on health care professionals, coordination and communication gaps, and compliance with privacy regulations. These challenges are often associated with inefficiencies and diminished care quality. We also examine how emerging artificial intelligence (AI) tools have the potential to enhance the management of patient information flow. Specifically, AI can boost interoperability across diverse health systems; optimize and monitor patient care pathways; improve information retrieval and care transitions; humanize health care by integrating patients’ desired outcomes and patient-reported outcome measures; and optimize clinical workflows, resource allocation, and digital tool usability and user experiences. By strategically leveraging AI, health care systems can establish a more robust and responsive digital information ecosystem, improving care coordination and patient outcomes. This perspective underscores the importance of continued research and investment in AI technologies in patient care pathways. We advocate for a thoughtful integration of AI into health care practices to fully realize its potential in revolutionizing care coordination.

## Introduction

**Figure 1:**
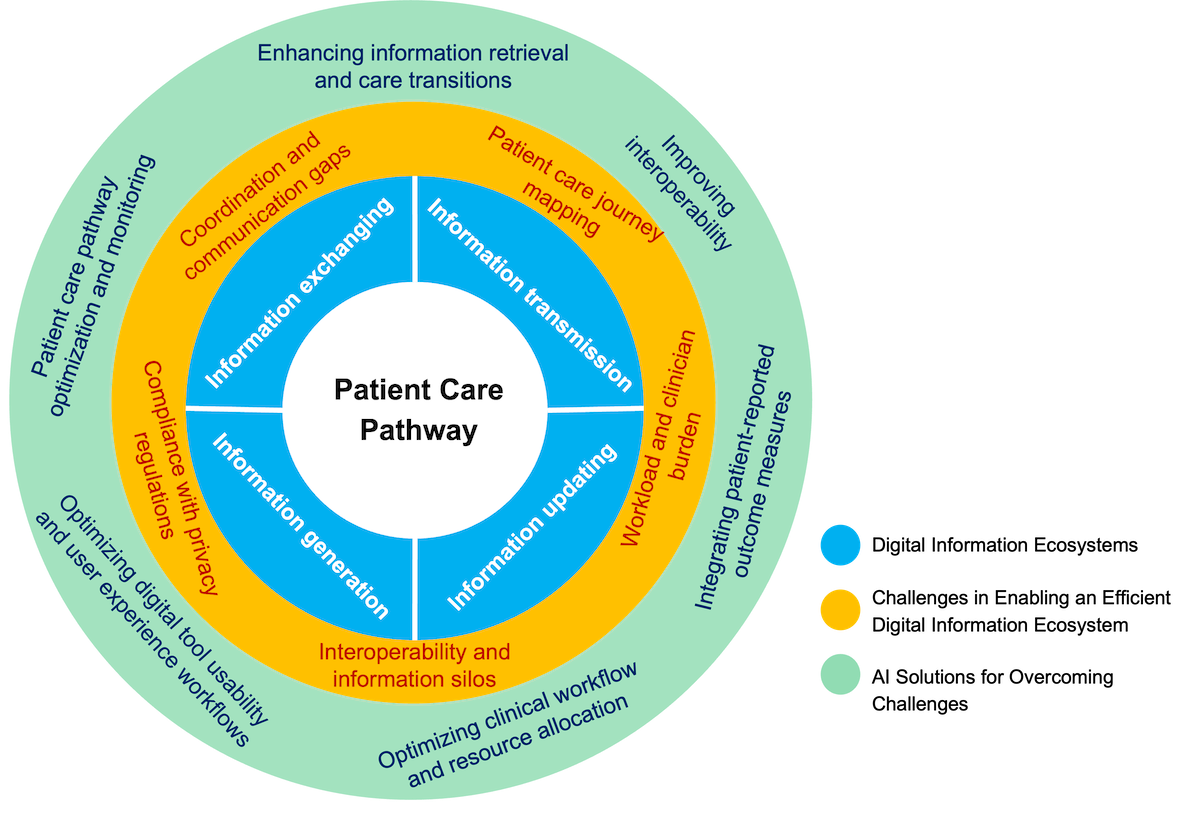
Comprehensive overview of the digital information ecosystem in modern care coordination and patient care pathways. The figure provides a detailed visualization of the digital information ecosystem, illustrating the flow of information generation, updating, transmission, and exchange along a patient’s care pathway. It encompasses all stages of patient care, including initial consultations, diagnostic tests, imaging, treatment interventions, ongoing monitoring, and referral to follow-up care. The figure highlights several key challenges that hinder the efficient management of the digital information ecosystem, such as interoperability issues, information silos, increased workload and clinician burden, coordination and communication gaps, patient care journey mapping, and the complexities of complying with privacy regulations. Potential artificial intelligence (AI) solutions are depicted as integral to overcoming these challenges, enhancing the efficiency of care coordination, and reducing the burden on clinicians. The figure underscores how AI can streamline processes, improve data flow and accessibility, and foster more effective coordination and collaboration across stakeholders. By integrating AI-driven tools and technologies, this ecosystem can achieve higher efficiency and more responsive patient care.

Care coordination, as defined by the US National Academy of Medicine, involves the organization of patient care activities and the deliberate distribution of information among stakeholders—including clinicians and patients—to ensure the appropriate delivery of health care services [1,2]. These services include diagnostics, treatments, care transitions, and the monitoring of patient self-reported measures and clinical outcomes. Efficient exchange and communication of information across a patient’s care pathway is fundamental to the care coordination process—a continuum that spans all activities from diagnosis through treatment and follow-up [3]. Today’s health care landscape relies heavily on digital technologies, such that it would benefit greatly from formally characterizing and optimizing the “digital information ecosystem” [4]. This ecosystem encompasses the complex network of digital technologies and protocols responsible for the generation, updating, transmission, and exchange of information among stakeholders along a patient care pathway. This perspective paper positions digital information ecosystems as the heart of modern care coordination, outlines the current challenges within this ecosystem, and explores how specific applications of artificial intelligence (AI) tools may enhance processes along the patient’s care pathway (Figure 1). By focusing on targeted AI solutions, we believe there are major opportunities to reduce the digital burden on stakeholders and enhance the efficiency of digital coordination, thereby improving the overall quality of health care delivery.

## Digital Information Ecosystem: Generation, Updating, Transmission, and Exchange

### Overview

The essence of care coordination lies in the seamless generation, update, transmission, and exchange of digital information, which supports both synchronous and asynchronous communications among stakeholders. Information generation corresponds to the creation of new data entries during the provision of health care services. There are numerous examples of information generation, such as the laboratory test results produced during diagnostic testing, or the diagnoses documented by health care professionals following patient evaluation. After generation, information updates occur, which involve modifications to existing health records to reflect new developments or changes in a patient’s condition. Examples of these activities include updates to progress reports, medication management, or any alterations made to previously recorded health information. The information transmission process pertains to the movement of information along specific pathways within the health care setting, such as prescriptions being transferred from physicians to pharmacists or care instructions moving from specialists to nurses. Finally, information exchange refers to the dynamic process where health information is not only shared but also appropriately interpreted and used across different health care systems and stakeholders. Exchange emphasizes the adherence to standards, protocols, or formats to ensure that the information is communicated accurately and meaningfully, supporting coherent and coordinated health care delivery.

An effective digital information ecosystem facilitates a comprehensive and timely understanding of a patient’s health, thereby informing clinical decisions and ensuring timely interventions. Given the complexity and extensive scope of the digital information ecosystem, we will simplify our introduction of this concept by focusing on electronic health records (EHRs) and their patient portal extensions. These platforms serve as prime examples of how digital tools facilitate the flow of information among stakeholders. To illustrate this further, we will explore specific scenarios involving the management of chronic diseases, demonstrating how information is exchanged and used within these platforms.

### Information Generation Along a Patient’s Care Pathway

Information about a patient’s health or their health care is generated at every key interaction point along a patient’s care pathway, from registration, initial consultations, and diagnostic tests to treatment interventions and ongoing monitoring. Throughout the care pathway for chronic conditions like diabetes, the generated information continuously informs care strategies. The flow of such information, as facilitated by digital platforms such as EHRs or patient portals, evolves with the patient’s condition, treatments, and responses, making it a dynamic and integral part of the care coordination process. This flow supports a range of care activities, ensuring that the right stakeholder has the right information at the right time to make informed decisions and provide effective care.

EHRs and patient portals play pivotal roles in generating health care information [5]. EHRs document a comprehensive range of data including diagnoses, procedures, laboratory test results, medications, orders, flowsheets, clinical notes, medical and surgical histories, allergies, and summaries of care during transitions [6,7]. These data can be composed to form the Continuity of Care Record [7], which supports clinical decision-making and ensures continuity of care. Integration of Continuity of Care Record into care coordination was associated, for example, with improvements in glycemic and lipid control for patients with diabetes [7]. Evidence suggests that EHR designs that store patients’ values, health goals, and action plans may strengthen the continuity and quality of care between patients and primary care team members [8].

Building on EHRs, patient portals can enhance patient engagement by allowing individuals to actively contribute to their care process and provide self-reported measures and feedback. These platforms can improve communication between clinicians and patients, increase medication adherence, reduce appointment delays, and enhance patient satisfaction [9,10].

### Information Updates and Transmission Along a Patient’s Care Pathway

Passing information from one care provider to another is an important process that involves accessing and updating patient records. Consider the case of a patient undergoing nephrolithotomy: important stages that rely on shared information include the initial diagnosis, presurgery assessments, the surgery itself, postoperative care, and routine follow-ups. When a diagnosis of kidney stone is confirmed, it is entered into the EHR along with a time stamp and user identification. Every subsequent access or modification of this diagnosis by health care professionals is similarly recorded with a new time stamp. This fine-grained tracking ensures that any changes in the patient’s condition or treatment strategy are attributable, illustrating how the diagnosis has been reviewed and adjusted by different health care providers.

The pathway that information takes generates detailed logs regarding which stakeholders accessed or modified the data [11]. Continuing with our example, after the nephrolithotomy, the surgical team may update the patient’s EHR with the details of the surgery, any medication administrations, and follow-up needs. These time-stamped logs identify the stakeholder making the modification and indicate the direction of the information flow—such as from a urologist to a primary care physician. This level of detailed tracking enables a clear audit trail, but also is useful in characterizing how medical decisions were made and can be refined [11].

### Information Exchanges Along a Patient’s Care Pathway

Information flows continuously through care pathways, frequently being exchanged among various nodes within the health system, including hospital departments and units, and diverse stakeholders. Each stakeholder involved in care, whether they are physicians, nurses, administrative staff, or patients, relies on the continuous exchange of data essential for their respective roles. For instance, physicians and nurses require immediate updates to make informed medical decisions, while administrative staff need accurate information for billing and compliance purposes. Patients are increasingly accessing their health data through patient portals to actively participate in their health care decisions.

Stakeholders engaging with EHRs or patient portals participate in asynchronous and synchronous information exchange. Ensuring that the correct information is delivered at the right time is crucial for the efficiency and safety of health care delivery. Delayed or inaccurate exchanges can compromise patient outcomes and increase the administrative burden on health care providers [12,13].

To address these challenges, electronic health information exchange (HIE) standards such as Health Level Seven (HL7) Fast Healthcare Interoperability Resources are crucial to implement and to be refined. These standards have the potential to ensure that every node in the health system involved in a patient’s care has access to up-to-date accurate and relevant data, enhancing the overall efficiency and quality of health care delivery [14,15].

## Challenges in Enabling an Efficient Digital Information Ecosystem and Effective Care Coordination

### Overview

**Figure 2:**
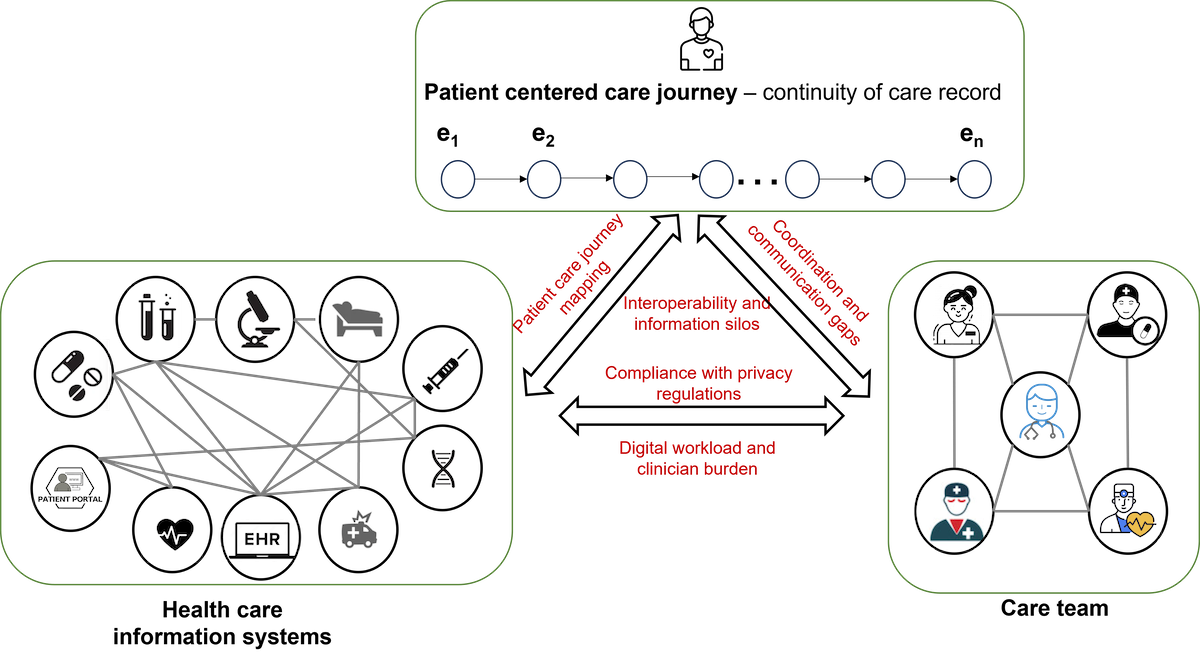
Challenges arising from interactions among the patient-centered care journey, health care information systems, and a network of health care professionals. The figure illustrates 3 interconnected components crucial to modern health care: the patient-centered care journey, health care information systems, and a network of health care professionals. The patient-centered care journey represents a sequence of care events along a patient’s health care trajectory. Health care information systems consist of various platforms containing diverse types of health and health care data about the patient. The network of health care professionals includes all the health care providers involved in the patient’s care journey, connected through professional collaboration. The interactions among these components give rise to several challenges in establishing an efficient information ecosystem and effective care coordination. EHR: electronic health record.

Despite significant advancements in digital technologies and interoperability, including the adoption of standards like Fast Healthcare Interoperability Resources and participation in national networks such as Care Everywhere, CommonWell, and Trusted Exchange Framework and Common Agreement [16], and the use of Direct Secure Messaging for communication between different EHR systems [17], challenges persist in managing the digital information ecosystem and care coordination efficiently. These challenges arise from the complex interactions that occur throughout the patient-centered care journey involving health care information systems and networks of health care professionals, as illustrated in Figure 2. Interoperability issues and information silos emerge when disconnected or incompatible health care information systems hinder seamless data exchange, leading to fragmentation of information. Difficulties in information retrieval, processing, and mapping of key care events impede the modeling of patient care pathways necessary for optimization and monitoring. Coordination and communication gaps can occur due to synchronous and asynchronous interactions through health care information systems, resulting in miscommunication and gaps in coordination among health care professionals. The extensive interaction with multiple health care systems increases the digital workload and contributes to clinician burnout, exacerbating the digital workload and clinician burden. Additionally, navigating regulatory requirements adds complexity to information sharing and coordination efforts, posing challenges in compliance with regulations.

### Interoperability and Information Silos

Interoperability in health care is crucial for the seamless sharing and use of information across various systems and platforms. Despite its importance, interoperability remains a significant challenge in digital coordination among health care providers. The landscape of formal HIE platforms in the United States is notably fragmented, characterized by a patchwork of efforts at federal, state, community, enterprise, and EHR vendor levels (eg, Epic Care Everywhere) [18]. As a result, only certain provider organizations are equipped to engage in HIE with others with whom they routinely share patients [19,20].

The use of EHR systems from different vendors, along with other medical devices, often exacerbates challenges, leading to ineffective communication and the creation of information silos within and across organizations [19,21]. These silos significantly hinder the process of coordinated care, as they prevent the efficient flow of crucial health data among the involved parties. This lack of streamlined data exchange continues to be a major barrier to achieving ideal interoperability that enables optimized health care delivery.

### Patient Care Journey Mapping

Patient care journey mapping refers to the process of creating a systematic representation of the sequence of care activities experienced by patients, thereby reflecting the continuity of patient care. For instance, Meyer [22] describes a patient with multiple chronic conditions who underwent posterior hip replacement surgery due to advanced avascular necrosis. Over the course of 21 months following the initial surgery, the patient received care across a wide range of settings, including 2 major surgical procedures and 9 care transitions. Without patient care journey mapping and monitoring, this patient and his family were unable to manage or plan medical appointments or self-care tasks. Mapping and monitoring the longitudinal patient journey could identify problems and gaps along the patient’s continuum of care, such as prolonged wait times, misalignment between medical needs and care teams, duplicated tests, adverse drug reactions, failure to follow-up, and fragmentation. Fung-Kee-Fung et al [23] showed that a lack of understanding of the patient care journey and the need for dynamic alignment of providers is associated with delays in timely lung cancer care.

Various approaches have been developed for mapping patient care journeys. The Customer Journey Mapping Language is a framework for customer journey analysis [24]. Additionally, data-driven pathway modeling, such as Process Mining, has been relied upon to build pathways by using existing EHR log data to display the actual patient pathway [25]. Conformance analysis in the context of process mining is one of the main forms of process mining [26-28], using a pre-existing process model and an event log. The pre-existing process model is often a protocol, guideline, or formally defined care pathway, and EHRs generally serve as the event logs.

The Unified Modeling Language provides a more system-centric modeling method, connecting stakeholders, tasks, and system intended functionality [29]. However, it is nontrivial to capture the entirety of the patient care journey due to the fact that not all care activities are documented in health information systems. Moreover, even when such information is documented, there are challenges with (1) information siloing, (2) differences in use behaviors and terminologies, and (3) a lack of integrated technologies that can perform information retrieval, processing, and mapping of key care events impede the modeling of patient care pathways, and generate a common language easily understood by involved stakeholders.

### Coordination and Communication Gaps

While EHR systems enable real-time access to data, retrieving relevant information efficiently remains a substantial challenge. EHR systems frequently lack direct communication tools that would allow stakeholders to discuss specific patient information. This deficiency can nontrivially impede effective interdisciplinary collaboration, as it complicates team members’ ability to access and discuss comprehensive patient data seamlessly, resulting in delay and inaccurate information. In one recent study, it was shown that the application of an EHR system could negatively affect team function, noting that while they support task-oriented and efficient communication, the system may shift attention away from the human needs of the care team [30]. Such observations underscore the double-edged nature of EHR utility in clinical settings.

Introducing features that allow direct annotation or discussion within patient records in the EHR could significantly enhance the clarity and speed of health care delivery. Such functionalities would ensure that all team members are well-informed and aligned in their clinical strategies, fostering a collaborative environment and improving health care outcomes.

### Digital Workload and Clinician Burden

EHRs and patient portals are designed to enhance information exchange and care coordination, yet often these tools end up increasing clinician burden due to their complex interfaces and inefficient workflows. For instance, physician-rated EHR usability was independently linked to the odds of burnout [31]. Specifically, each 1-point increase in the System Usability Scale score was associated with a 3% decrease in the odds of experiencing burnout. Thus, despite their intended purpose, EHRs frequently require extensive data entry and navigation, which diverts attention away from patient engagement and care and can contribute to physician fatigue and burnout [32].

This problem becomes particularly pronounced in settings that manage complex care pathways, such as treatment for patients with chronic kidney disease. In these scenarios, timely and accurate information exchange is critical, and the demands placed on clinicians by digital tools can significantly impede the efficiency and effectiveness of care delivery.

### Compliance With Regulations

Research shows that while patients are generally open to sharing their protected health information (PHI) to support collaborative care, they continue to have concerns about the privacy of their personal health information during the sharing process [33]. Addressing these concerns is essential. By enhancing security measures and increasing transparency on PHI management, health care providers can build greater trust among patients. This improves the flow of information and ensures that patients understand and feel reassured about the protections in place to safeguard their privacy, which is crucial for maintaining compliance and integrity within the digital information ecosystem.

As digital information traverses the care pathway, ensuring adherence to regulations like the Health Insurance Portability and Accountability Act (HIPAA) becomes increasingly complex. In one survey of surgeons, a non-trivial proportion used text messaging to communicate PHI [34]. Yet HIPAA stipulates that text messaging of PHI is only permissible if the patient has initiated communication via SMS or has requested confidential communications through this medium. It is crucial for stakeholders to implement robust security measures and establish clear protocols to prevent any breach of privacy during electronic communications, ensuring compliance with HIPAA and other federal or state-specific laws.

## AI Solutions for Overcoming Challenges in the Digital Information Ecosystem

### AI in Improving Interoperability

As the health care industry continues to enhance its health information technology connectivity infrastructure, AI has contributed to integrating and harmonizing data across multiple systems [35-37]. AI significantly advances semantic interoperability—the ability of different systems to exchange data accurately with a common format and meaning [38,39]. This is particularly evident in AI’s capacity to extract meaningful information or infer actionable insights from structured and unstructured data sources, thereby improving data exchange across the care continuum.

A key aspect of AI’s role is the conversion of free-text information into structured data, which is invaluable for creating comprehensive patient records that are compatible across different systems. This enhances data fluidity and accessibility, making it easier to share information across systems. Natural language processing (NLP)–driven AI tools are essential in transforming unstructured clinical text into a structured format. A recent study using Stanford Open Information Extraction demonstrated how AI can structure diagnostic information from free-text pathology reports [40]. The AI model effectively captured complex diagnostic entities and their relationships, converting this information into knowledge graphs. The resulting structured data showed a high semantic similarity to the original reports (mean weighted overlap of 0.83), illustrating the model’s ability to preserve the integrity of the original clinical information.

Moreover, AI can integrate structured and unstructured data to generate actionable insights. An AI algorithm—an ensemble model combining latent Dirichlet allocation with gradient-boosted trees—was developed to predict and diagnose sepsis [41]. By leveraging structured data and unstructured clinical notes, the AI model achieved high predictive accuracy (area under the receiver-operating characteristic curve of 0.94, sensitivity of 0.87, and specificity of 0.87) up to 12 hours before the onset of sepsis. This model demonstrated the potential to increase early detection of sepsis by up to 32% and reduce false positives by up to 17% compared with physician predictions.

In addition to its clinical applications, AI-powered tools can streamline administrative tasks in health care, such as scheduling appointments, managing billing, and processing insurance claims [42-44]. Improved interoperability between different administrative systems leads to more efficient health care operations, reducing the administrative burden, and allowing health care professionals to focus on patient care rather than paperwork.

### AI in Patient Care Pathway Optimization and Monitoring

AI has been increasingly applied to optimize patient care pathways by improving data integration, standardization, and predictive modeling. These AI-driven solutions facilitate better coordination among health care professionals, reduce delays in treatment, and enhance overall patient outcomes. One notable example is the implementation of a learning health system by The Ottawa Hospital, which connected data across silos to redesign regional lung cancer diagnostic pathways [23]. By integrating 12 major processes—including referral, review, diagnostics, assessment, triage, and consultation—the hospital significantly streamlined the patient care journey resulting in a reduction in the median time from referral to initial treatment by 48%, dropping from 92 to 47 days. This demonstrates how data integration and process optimization can substantially improve the efficiency of patient care. Enhancing data quality and transforming unstructured data into actionable insights are also helpful for effective care pathway management. In a study by Levine et al [45] who developed NLP annotators to transform unstructured EHR text into structured attributes for patients with stage III breast cancer, the researchers enabled a clearer understanding of the clinical course by compiling events in the patients’ journeys to create timelines. The NLP system showed high agreement with manual chart reviews, differing in only 6 out of 171 data elements, thus validating its accuracy and utility in clinical settings.

Building upon data integration and standardization, AI can automate the generation of care pathways aligned with clinical guidelines. In pediatric oncology, one study demonstrated how knowledge engineering methods alongside AI planning and scheduling techniques could automatically generate care pathways from computer-interpretable clinical practice guidelines [46]. This approach coordinated the activities of health care professionals involved in patient treatment, adapting care plans based on patients’ current health conditions and deviations from expected progress. The system effectively handled exceptions, producing suitable care plans in 71.88% (23 out of 32) of the executed care pathways during simulation. Advancing further, AI models can predict patient health trajectories and outcomes, enabling proactive care planning. The development of a long short-term memory–based tool called DeepCare exemplifies this capability [47]. DeepCare represents care episodes as vectors and models patient health state trajectories by aggregating historical and current health data through multiscale temporal pooling. This information is used in a deep dynamic neural network that estimates future outcomes, handles irregularly timed events, and models medical interventions. When applied to datasets related to diabetes and mental health, DeepCare demonstrated improved prediction accuracy in outcomes, showcasing its potential in predictive analytics. Additionally, simulation models can aid in understanding complex patient care pathways and the potential outcomes of interventions. Researchers created a digital simulation to model the clinical pathways of critically ill patients by transforming causal and associative relationships into structured expert rules [48]. Depicted in a directed acyclic graph format and stored in a graph database (Neo4j), these rules drove a simulation app enabling users to simulate patients’ state trajectories over time and test the effect of different interventions on outcomes, facilitating decision-making in critical care settings.

Collectively, these examples illustrate the great potentiality of AI in patient care pathway optimization and monitoring. By automating the generation of care pathways, adapting to patients’ changing health statuses, and predicting future outcomes, AI can bridge and integrate information from various health information systems. This integration facilitates the creation of a common language easily understood by the stakeholders involved. Moreover, AI enables the prediction of steps in the patient journey and the simulation of care pathways with different parameters, identifying the most effective ones to improve patient outcomes. However, the development and implementation of such AI technologies are still in their early stages, indicating a need for continued research and refinement in this area.

### AI in Enhancing Information Retrieval and Care Transition in Patient Care Pathways

In modern health care, despite the prevalence of digital technologies, stakeholders—including health care professionals and patients—face challenges in effectively retrieving key health information to improve responsiveness along a patient-centered care journey [49-53]. The heavy workload associated with managing vast amounts of heterogeneous information can lead to gaps or inaccuracies in understanding patient health conditions complicating clinical decision-making and affecting patient outcomes. AI applications that successfully retrieve, code, and summarize key health information along a patient care journey to make predictions or decisions demonstrate the transformative potential of these technologies.

Care transitions are particularly vulnerable phases where inconsistencies in handoff records can lead to adverse events. AI has the potential to automate and verify handoff records, such as medication lists across care transitions, ensuring accuracy and continuity of care [54]. Inefficient care summaries can also create gaps in care, especially during transitions between different services. AI-generated summaries that automatically highlight critical care points can improve the effectiveness of these transitions [55,56]. A recent simulation study assessed the effectiveness of NLP combined with a support vector machine to automatically code handoff communication content and behaviors during transitions from the operating room to the intensive care unit [57]. The coded communication content included patient information, anesthesia details, surgical data, nursing information, equipment and technology used, patient transfer, and the professional environment. The communication behaviors analyzed encompassed information giving, seeking, and verification, as well as assessment, planning, and decision-making. The support vector machine model achieved coding accuracy scores of 74.2% for communication content and 54.8% for communication behaviors. A qualitative study involving semistructured interviews with 11 nurses explored the potential uses of AI in perioperative nursing handoffs, which heavily rely on structured checklists [58]. The findings suggest that AI highlighted critical care points and identified issues that could be incorporated into handoff discussions and team communications. AI-estimated elevated risks might prompt patient re-evaluation, and AI-highlighted important data could add value to nursing assessments.

The AI-Pathway Companion is another example of the potential for using AI to manage vast amounts of data, including radiology, pathology, genetics, and laboratory results from hospital information systems and picture archiving and communication systems [59]. The AI-Pathway Companion Prostate Cancer VA10B, approved for use in Europe as a medical device, leverages NLP to match the data available for an individual patient with prostate cancer guidelines from the European Association of Urology and the National Comprehensive Cancer Network. It identifies the recommended treatment approach that suits the patient’s current treatment status [59]. The companion automatically shows the patient’s location in the pathway and recommends the next steps, including any missing information required. The implementation of this software significantly reduced consultation preparation times in prostate cancer management and effectively improved the decision-making process and customer satisfaction. Another example is an e-pathway embedded into the EHR [60], such as a Head and Neck Oncology e-pathway that was developed to categorize by tumor localization and tumor stage. For every phase of the care process, agreements among involved health care professionals from different specialties were made regarding when and who should perform specific tasks, record specific information, determine required orders for providing optimal care, and schedule when these orders should be placed. The e-pathway aimed to automatically capture all relevant discrete data during the care process, eliminating the need for manual data collection. In this project, the team used smart phrases, autotexts, and content-importing technology to automate and standardize documentation of every phase in the pathway, including the initial oncology consultation, multidisciplinary tumor board meeting, diagnostic result consultation, follow-up consultation, and prefilled, standardized order sets. The study showed that total EHR time in initial oncology consultations was significantly reduced by 3.7 minutes—a 27% decrease compared with before using the e-pathways.

### AI in Humanizing Health Care by Integrating Patient-Reported Outcome Measures

The integration of patient-reported outcome measures (PROMs) into AI-enabled processes can further help AI to personalize and potentially humanize health care [61-63]. AI algorithms are increasingly capable of processing PROM data in real-time and suggesting possible adjustments to care plans. In a review of 152 trials conducted before September 2022, it was shown that AI-enabled tools, including machine learning, reinforcement learning, neural networks, and NLP incorporating PROMs were frequently evaluated in areas like musculoskeletal health, mental health, and metabolic conditions such as diabetes [64]. Notably, 24 of these trials specifically evaluated AI models that used PROMs as input, highlighting the growing trend of integrating patient perspectives and personal outcome desires into AI-driven health care. In one significant study, researchers developed a shared decision-making tool using machine learning to generate personalized predictions of risks and benefits for total joint replacement surgery based on PROMs and clinical data [65]. This tool was evaluated in a randomized controlled trial, where its effect on decision quality and patient outcomes was assessed. Similarly, in the field of rehabilitation, machine learning models relying on commonly used algorithms like linear regression, Random Forest regressor, Extra Trees regressor, Linear Support Vector Regression, and Kernel Ridge with a polynomial kernel were trained on clinical and PROM data from over a thousand patients. This model successfully predicted the likelihood of rehabilitation success, allowing clinicians to tailor treatment plans from the start of therapy [66].

While AI-enabled smart devices, clinical decision support systems, and chatbots have shown potential in retrieving, summarizing, coding, and analyzing clinical and PROM data to personalize and enhance treatment effectiveness [62,63], their adoption is not without challenges. These include fragmented data collection processes, the scarcity of comprehensive clinical and PROM datasets, and the rigorous monitoring and validation requirements for AI systems. Moreover, many studies integrating clinical, PROMs, and AI often exhibit suboptimal design [62,67].

### AI in Optimizing Clinical Workflows and Resource Allocation Along the Patient Care Journey

There is a growing demand for AI models that can enhance clinical workflow and resource allocation across various stages of the patient care journey—such as registration, triage, diagnosis, and treatment. A qualitative study conducted in 2 German hospitals confirmed the potential of AI-based processes, specifically AI-driven text recognition and monitoring, to alleviate overcrowding in emergency departments [68]. By automating data entry and monitoring patient flow, AI can manage patient influx more effectively, reduce wait times, and improve patient satisfaction. Another study developed machine learning models to automate scalp electroencephalogram seizure tracking, supporting a digital care pathway for epilepsy [69]. The results showed that automating the diagnostic process within the digital care pathway could significantly reduce the time needed to diagnose epilepsy. This acceleration in diagnosis not only improves patient outcomes but also optimizes the use of health care resources by reducing the burden on medical professionals.

Recent advancements in AI, such as the release of ChatGPT-4, demonstrate its potential to optimize clinical workflows and reduce clinician burden. For example, integrating large language models (LLMs) into clinical workflows to draft responses to patient inbox messages has been shown to improve clinician well-being [70]. Additionally, in one study focusing on OpenAI’s ChatGPT models, it was shown that AI assistance not only reduced physician workload but also enhanced the consistency, informativeness, and educational value of responses to patient messages [71].

These benefits highlight AI’s potential to transcend beyond traditional tools, such as dashboards, by adding an adaptive, real-time optimization layer. Unlike static dashboards that visualize a patient’s journey through the health care system, LLM-driven AI can continuously refine and update patient journey maps, ensuring that health care providers have the most accurate and timely information. This ability to actively process and synthesize complex information sets AI apart, making it an asset for improving clinical workflows and patient outcomes.

Another significant challenge in clinical workflow is the complexity of accessing and managing patient care-related digital information, which contributes to EHR workload [30]. LLMs have the potential to proactively deliver the required information to the correct clinicians at the right time, eliminating the need for extensive searches or queries [72]. By providing customized information displays tailored to the needs of each stakeholder group, AI has the potential to enhance efficiency and improve user satisfaction. For example, AI-generated tools could include context-aware messaging systems that automatically link discussions to relevant patient data, or intelligent summarization algorithms that quickly provide overviews of patient status changes [73]. This could be particularly useful in environments like intensive care units, where real-time monitoring and customized insights can significantly affect strategic planning and patient outcomes.

In the realm of medical imaging where many Food and Drug Administration (FDA)–approved AI technologies exist [74], AI, particularly deep learning, is already assuming a pivotal role in optimizing clinical workflows. The advanced computational capabilities of deep learning enable it to analyze complex features in imaging data, thereby improving image acquisition, providing real-time assessments of image quality, and aiding in objective diagnoses [75]. For instance, convolutional neural network-powered ultrasound systems can evaluate multimodal data, guide sonographers, and offer objective qualifications and diagnoses [76]. This not only supports clinical decision-making but also enhances workflow efficiency and reduces costs by minimizing dependency on operator expertise. A practical example is the health care enterprise radiology AI workflow for imaging, which streamlines the process from imaging examinations to report generation, significantly reducing radiologists’ workload [77]. The deployment of an AI system with integrated components—ranging from image delivery and quality control to error correction and performance monitoring—demonstrates how AI can improve the efficiency of radiology workflows.

### AI in Optimizing Digital Tool Usability and User Experience Workflows

Beyond clinical workflows, AI’s applications extend to improving user experience (UX) in digital health tools. AI-based tools that monitor user behavior, such as mouse tracking and click models, can infer a user’s needs and optimize interface designs accordingly. For instance, a deep neural network model called the “click model” was developed to simulate user interactions within a mobile app, leading to an optimized design that improved efficiency, reduced errors, and increased user satisfaction [78].

These AI-driven enhancements in UX are directly linked to the broader context of health care operations, where digital tools play a role in coordinating asynchronous and synchronous communication along a patient’s care journey. By monitoring the use of these tools and identifying inefficiencies, AI can enable health care providers to optimize prompt use, address barriers to information exchange, and mitigate potential clinician burdens and adverse events [79]. Studies have shown that workload, as measured by EHR audit logs, is linked to physician burnout and responsiveness to patient inquiries, underscoring how AI-driven insights can identify these issues and address them [80]. AI’s ability to analyze large-scale, granular, and continuously updated data allows it to identify workflow bottlenecks and inefficiencies that might be overlooked in traditional usability studies [81-83]. For example, AI can identify features within the EHR interface that are frequently accessed but inefficiently positioned, suggesting redesigns that reduce the number of clicks and screen transitions required for routine tasks [84,85]. While traditional usability studies can highlight areas for improvement based on observed user behavior, AI can go further by tracking every interaction across thousands of user sessions, revealing inefficiencies that may occur in specific contexts or evolve over time.

Furthermore, AI has the potential to uncover non-intuitive patterns that might not be apparent even to experienced usability experts. For example, an AI might discover that certain combinations of actions consistently lead to longer task completion times, identifying areas for redesign that were not previously considered [86].

Last, the growing interest in applying AI to support UX workflows highlights AI’s versatility in health care. UX design heavily depends on human creativity and input, aiming to meet end user requirements and expectations. It is a user-centered, iterative process where piloting, testing, and refining are key elements. AI offers new opportunities for human-AI collaboration in creative tasks, enabling cooperation in different phases of the UX design process. Various industry surveys indicate that UX professionals view AI-driven features in design tools positively, seeing AI as a creative partner that can iterate and innovate alongside human designers [87]. A critical step for AI is automating the transformation of a mock-up of a graphical user interface (GUI) into code. Such automation can facilitate the creation of adaptive interfaces that dynamically evolve based on changing user requirements. One study developed a machine learning-based tool called ReDraw [88], which integrates software repository mining, automated dynamic analysis, deep convolutional neural networks, and k-nearest neighbors to automate the transformation of GUI mock-ups into code. This enables accurate prototyping of GUIs. Interviews with industry practitioners illustrate ReDraw’s potential to improve real-world UX workflows.

While most AI-enabled systems supporting UX work focus mainly on GUI elements, design activities that involve more “design thinking,” such as user interviews and user testing, are particularly helpful for designers. LLMs have shown remarkable capabilities in creative tasks like idea generation, providing exceptionally human-like responses. A study [89] examined human-LLM interaction in UX designers’ practices of incorporating ChatGPT into their professional design activities from a communication perspective, demonstrating the potential of ChatGPT in creating more productive UX tools. Another study [90] showcased a proof-of-concept implementation where users submit textual descriptions of their desired website features, prompting the LLM to produce corresponding HTML and CSS code and even provide recommendations for UX design. This approach allows users to update specific parts of the code or add new components generated by the LLM, rather than rewriting the entire document and including unnecessary code revisions, thereby improving the creativity and efficiency of UX design.

AI-human collaboration in enhanced user interface design has the potential to improve operational efficiency and reduce the learning curve for stakeholders, including health care professionals and patients.

## AI-Based Tools: From Evidence to Practice

Transitioning from evidence to practical application involves multiple steps. Before fully implementing new tools or features developed through AI and big data analyses, it is essential to conduct pilot and clinical testing in controlled environments. This preliminary phase helps to evaluate the effect of these innovations on coordination efficiency and clinician workload.

Engaging the stakeholders—not just clinicians but also patients, IT professionals, and administrative staff—in the redesign process is crucial to ensure that the changes effectively address real-world needs and increase the likelihood of their adoption [36,91]. For example, AI tools designed to summarize and maintain key information along a patient care journey need to be intuitive and responsive. The stakeholder can be involved in shaping these tools to include various anticipated features, such as alerts for updates to critical information, ensuring that these enhancements are useful and user-friendly.

Establishing mechanisms to continuously collect feedback from all stakeholders and perform iterative updates based on actual use is vital. An ongoing feedback loop will allow for the refinement of AI tools, so they better meet the evolving needs of health care professionals and patients. By adapting AI tools based on direct input from end users, health care systems can optimize the functionality and influence of these technologies, ensuring they effectively support clinical decision-making and patient care.

## Limitations and Considerations in the Design of AI-Based Tools

One of the significant limitations of AI-based tools is the potential for inherent biases, which can arise from the data on which these systems are trained. If the training data are not representative of diverse patient populations, or the specific population they will be applied to, AI algorithms can develop biases that lead to unequal treatment outcomes. These biases could potentially affect diagnostic accuracy, treatment recommendations, and patient management, disproportionately influencing certain demographic groups and leading to inequitable care [92-95].

While AI has already demonstrated its potential to enhance accuracy in some areas of health care, such as medical imaging, addressing the challenges in other fields—like mental health—requires ongoing research and development. Their accuracy remains a critical concern, particularly for multimodal data, where a lack of comprehensive frameworks can affect the performance, reproducibility, and transparency of AI algorithms. Continuous monitoring and updating of the algorithms are essential to maintaining accuracy as new data become available. By focusing on improving data quality, model transparency, and rigorous validation, AI can become a more trustworthy and effective tool across all areas of health care [94].

As AI systems rely on large volumes of data to function optimally, this dependence raises additional concerns about privacy and security, particularly regarding sensitive patient information [96]. Ensuring compliance with regulations like HIPAA in the United States or the emerging AI Act in the European Union will be critical. AI tools must be designed with robust security measures to protect patient data and prevent unauthorized access.

## Conclusions

Addressing the challenges in managing information flow—including data generation, updating, passing, and exchange along the patient care pathway—is critical to enhancing digital coordination. AI offers promising solutions by boosting the efficiency of the digital information ecosystem, improving interoperability and clinical workflow, optimizing patient care pathways, enhancing digital tool usability and UX workflows, reducing clinician burden, and ensuring privacy compliance. Moreover, initial practical implementations in organizations equipped to effectively implement and maintain these technologies are showing significant promise. As more health care systems begin to incorporate these technologies into their operational and clinical workflows, a richer picture will emerge, providing clearer demonstrations of their effectiveness. We anticipate that this growing body of evidence will inform continuous improvements in health care delivery systems. As such, we advocate for strategic investments in these technologies, along with ongoing evaluation and adaptation, to advance digital collaboration in health care and ultimately enhance the quality and outcomes of patient care.

## Abbreviations

AI: artificial intelligence
EHR: electronic health record
FDA: Food and Drug Administration
GUI: graphical user interface
HIE: health information exchange
HIPAA: Health Insurance Portability and Accountability Act
HL7: Health Level Seven
LLM: large language model
NLP: natural language processing
PHI: protected health information
PROM: patient-reported outcome measure
UX: user experience
